# Effect of nutrition awareness on utilization of Orange Fleshed Sweetpotato among vulnerable populations in Kenya

**DOI:** 10.1007/s12571-022-01326-4

**Published:** 2022-12-19

**Authors:** Chalmers K. Mulwa, Simon Heck, Joyce Maru, Josephine Mwema, Hugo Campos

**Affiliations:** 1 International Potato Center, Sub-Saharan (SSA) Africa Regional Office, Nairobi, Kenya; 2 World Food Program, Nairobi, Kenya; 3 International Potato Center, Lima, Peru

**Keywords:** Vitamin A deficiency, Nutrition awareness, Orange fleshed sweetpotato, Kenya

## Abstract

Malnutrition continues to affect many vulnerable populations worldwide, with the majority of these residing in developing and underdeveloped countries. This problem has been exacerbated by the changing climate and more recently by the COVID-19 pandemic. Urgent efforts geared towards enhancing sustainable production and value chains of nutritious foods to ensure access to healthier diets are therefore critical. A recent partnership between the World Food Programme and the International Potato Center to enhance utilization of biofortified crops in fragile environments in Kenya is a step in this direction, aimed at improving the diets of households at risk of hunger and malnutrition. This study sets out to provide early evidence on the potential impacts of the interventions spearheaded in this partnership, together with lessons for further scaling efforts. Using household level data, the study adopts an impact evaluation framework to understand the effect of nutrition awareness through the dissemination of information on Vitamin A deficiency, on the utilization of orange fleshed sweetpotato, a biofortified crop rich in Vitamin A. Results show positive and significant effects of nutrition awareness on utilization of the orange-fleshed sweetpotato. Several factors were also identified as key to determining the exposure to nutrition awareness, including proximity to markets and extension agents, gender, and education levels. For widespread and inclusive adoption and utilization of orange-fleshed sweetpotato, out-scaling efforts need to consider these determinants in designing interventions aimed at raising nutrition awareness, as a key entry point to enhancing utilization of orange-fleshed sweetpotato.

## Introduction

Vitamin A deficiency (VAD) continues to afflict many segments of the world’s population, most of these residing in Low- and Middle-Income Countries. It is estimated that VAD accounts for over 600,000 deaths each year globally for children under five years of age, and is also associated with severity of diarrhoea among children, and anaemia and night blindness among pregnant women (Hotz et al., 2012a, b). Scaling-up the adoption and utilization of biofortified crops is seen by many as a sustainable way of fighting the twin problems of food and nutritional insecurity (Van Der Straeten et al., 2020). Yet while concerted efforts have led to the development of biofortified crops capable of fighting hidden hunger, adoption and utilization of these remain low, typical of other agricultural technologies in sub-Saharan Africa (SSA), due to various demand- and supply- side constraints (see Ruzzante et al., 2021 for a review). This calls for demand creation strategies, as well as efficient delivery models for disentangling supply- side constraints, for enhanced biofortification at scale (Foley et al., 2021).

Orange fleshed sweetpotato (OFSP) has been shown to substantially improve Vitamin A intake among children below the age of five, and that of pregnant and lactating women (Girard et al., 2017; Hotz et al., 2012a). The International Potato Center (CIP) and partners in Africa, Asia and Latin America have developed more than one hundred locally adapted OFSP varieties and have promoted their dissemination through integrated delivery models including seed system, agronomic, post-harvest and demand creation interventions (Low & Thiele, 2020). These efforts have resulted in the adoption of OFSP by more than six million smallholder farmers (Heck et al., 2020). Based on its proven nutrition efficacy and the effectiveness of delivery models, OFSP is increasingly being considered as a nutrition-sensitive technology in humanitarian programming in regions without prior history of sweetpotato cultivation and with scant awareness of vitamin A deficiency and mitigating strategies. In this context, it is important to understand the relative role of demand-side interventions, such as awareness creation, for the adoption and utilization of OFSP among target populations, and how these interventions might be best combined with initial supply-side interventions to kick-start sustained adoption. Several studies have pointed to the importance of nutrition awareness creation for the acceptance and active utilization of biofortified crops, including OFSP (Adekambi et al., 2020; Shikuku et al., 2019).

In order to promote the utilization of OFSP as a part of sustainable food systems and livelihoods in ASAL regions of Kenya,CIP and the World Food Programme (WFP) have been conducting interventions in the arid and semi-arid lands (ASAL) in Kenya since mid-2020. The partnership aims to exploit individual institutional strengths, with the former being an agricultural research center of excellence while the latter is the world leading humanitarian organization with demonstrated capacity to reach the most vulnerable, to achieve the mutual goals of combating food insecurity and malnutrition among society’s most vulnerable groups. Several interventions are included in this CIP-WFP partnership, key among these being raising households’ awareness of VAD and the potential of OFSP in increasing Vitamin A intake (hereafter referred to simply as “nutrition awareness”). This study aims to give early evidence on the effect of raising this nutrition awareness on utilization of OFSP, as a way to combat VAD in the region, through enhanced vitamin A intake.

The study builds on extant literature on the importance of exposure to technologies for successful adoption (Adekambi et al., 2020; Diagne & Demont, 2007; Simtowe et al., 2016) and uses an impact evaluation framework to understand the effect of interventions aimed at raising nutrition awareness on OFSP utilization, and the drivers of exposure to nutrition awareness. This is important in assessing whether ongoing interventions are achieving intended results, as well as in guiding design of future interventions for higher and inclusive impacts. While the interventions pursued through the partnership take different forms, the focus on nutrition awareness is premised on the assumption that this is an enabling condition towards OFSP utilization. It is also unreasonable to assume that other interventions would be pursued in isolation without nutrition awareness. In this paper, OSFP utilization is defined as the various ways that households consume OFSP (i.e., either as boiled roots at home, restaurants or from food vendors OR as puree for small children) by both producing and non-producing households who may access OFSP roots through the market or food aid.

The study contribution is two-fold; by focusing on consumption rather than adoption by sweetpotato producers, the study brings forth the role of markets in OFSP utilization, which is particularly important in this case since a large section of the population in ASALs are pastoralists. Secondly, using well-known impact evaluation methods, the study offers early evidence on the importance of novel institutional partnerships in achieving impact at often hard to reach vulnerable population segments.

## Background to CIP-WFP interventions in the study area

The WHO defines marginal vitamin A deficiency as a plasma or serum retinol concentration of between 1.05 μmol/L and 0.7 μmol/L in adults and children, with Vitamin A deficiency being a concentration of less than 0.7 μmol/L (WHO, 2009). In Kenya, the prevalence of vitamin A deficiency and marginal vitamin A deficiency among children less than five years old is 4.5% and 24.2%, respectively (Kenya demographic Health Survey, 2014). This situation is dire in fragile areas like the ASALs in the northern and eastern parts the country, where access to food is greatly curtailed due to unfavorable production conditions and low accessibility. Climate resilient livelihoods are being adopted including the production of OFSP which contributes to better nutrition and diversity in agricultural production given the crop’s resilience to climatic stressors.

More recently, humanitarian agencies including WFP in their journey towards sustainable food systems and resilient livelihoods have started prioritizing nutritious value chains including OFSP in their food systems and livelihoods programming through partnering with institutions like CIP, with an initial dual focus on OFSP production by vulnerable households and utilization of OFSP puree for young child feeding at household level. The pursued interventions in this CIP-WFP partnership have adopted a systems approach with the goal of enhancing OFSP utilization at the household level. Production at the household level is supported through establishing seed supply chains; local farmers are trained as ‘Decentralized Vine Multipliers’ (DVMs) and are linked to national organizations such as the Kenya Plant Health Inspectorate Service (KEPHIS) and the Kenya Agricultural & Livestock Research Organization (KALRO) for accessing clean seed for multiplication. Producing households are then linked to these DVMs, creating a supply of affordable OFSP planting material at the local level, while public extension workers are also trained on OFSP production and good agricultural practices for onward training to producing households. On the demand-side, several interventions are supported that aim to raise nutrition awareness. Such interventions include training of traders involved in sweetpotato marketing on OFSP as a high value nutritional crop and providing nutrition information to expectant and breastfeeding mothers, as well as providing them with the “Healthy Baby Toolkit (HBT)”, a CIP invention aimed at providing guidance to mothers on the preparation of OFSP puree for supplementing young children’s diets.

In this study we focus only on the demand-side interventions, as listed above, that are aimed at raising nutrition awareness. While other system-wide interventions like supporting sustainable seed distribution systems, linking producers to the market, and market support, are important in enhancing OFSP utilization at scale, we view nutrition awareness as an important initial enabling condition for household utilization of OFSP. Interventions aimed at disentangling supply-side constraints can achieve more impacts in an environment where targeted households have nutrition awareness and therefore more receptive to OFSP. The study therefore focuses on nutrition awareness as a crucial initial intervention in introducing OFSP in a region with low exposure to the crop, and where nutrition information is scarce.

## Conceptual framework and estimation strategy

### Conceptualization

Our study’s main objective is to understand how interventions aimed at raising nutrition awareness among populations living in ASAL regions of Kenya affect the utilization of OFSP among these populations, and the attribution of this to CIP-WFP interventions. To enhance utilization of OFSP in the study area, CIP-WFP interventions have adopted a market systems approach where biofortification goals are sought not only for producing households, but other value chain actors including traders and consumers. Based on this, our study departs from others that purely focus on the adoption of technology (see for example Adekambi et al., 2020; Diagne & Demont, 2007; Dimara & Skuras, 2003) to focus on utilization of OFSP, which is a composite variable of consumption by OFSP producing and non-producing (through market participation) households. While agro-pastoralism is predominant in these regions, a significant number of households are full pastoralists (Nyariki & Amwata, 2019), and the only way to achieve OFSP utilization at scale is through market purchases. Data from this study shows that about 54% of the interviewed households sourced sweetpotato from the market, with only about 56% having grown the crop in the previous two years (note that producing households may participate in markets through both selling and purchasing sweetpotato). This reinforces the rationale of our strategy to focus on OFSP utilization, as defined above, and not just OFSP adoption.

Unlike studies addressing similar problem to ours (see for example Adekambi et al., 2020; Diagne & Demont, 2007; Simtowe et al., 2016), our definition of “awareness” goes beyond the mere knowledge of the existence of a technology; instead, we define awareness of OFSP as the knowledge of its existence, including the potential nutritional benefits as a biofortified technology that can combat Vitamin A deficiency (VAD). This definition is more befitting in our case given the interventions led by the CIP-WFP partnership in raising awareness about VAD in the study areas and promoting utilization of OFSP as a potential remedy. Further, while such studies treat awareness of technology as trivial and rather focus on what informs adoption decisions, we posit that this first initial stage of awareness is critical to utilization of OFSP, typical of relatively new technologies (Dimara & Skuras, 2003). Understanding drivers to nutrition awareness is therefore important in this study, which is valuable in informing ongoing efforts to out-scale OFSP utilization in the intervention areas.

There are several challenges in estimating the effect of nutrition awareness on OFSP utilization. First, targeted areas for the CIP-WFP interventions aimed at raising nutrition awareness may benefit from intervention design bias, ranging from perceived state of malnutrition, physical accessibility, spatial spread of change agents (for example, clinics for training pregnant women and young mothers on nutrition awareness), among other factors. This may result in some systematic differences between areas reached by nutrition awareness interventions and those that are not, resulting in only a subset of the targeted population being exposed to the treatment, a phenomenon otherwise known as non-exposure bias (Atanu et al., 1994; Diagne & Demont, 2007; Dimara & Skuras, 2003). Secondly, diffusion of nutrition awareness across population of interest may differ systematically given factors like individual attitudes towards risk, self-interest and health consciousness, as well as other binding constraints like education levels, social capital, and proximity to change agents, etc. This gives rise to a self-selection problem in analyzing treatment effects in studies using observational data (Gitonga et al., 2020; Mulwa et al., 2021).

While randomization in well-designed experimental studies can overcome such challenges as described above, this is not possible in observational studies such as ours. The key challenge is the identification of a true counterfactual, in the presence of non-randomness, for consistent intervention impact estimation. Several approaches abound in the empirical literature for the estimation of treatment impacts in the presence of non-randomness in the treatment (Asfaw et al., 2018; Gitonga et al., 2020; Kassie et al., 2015; Mulwa & Visser, 2020; Terza et al., 2008). The endogenous switching regression (ESR) method has been extensively used in studies such as ours to correct for not only observable confounders but also hidden bias (Asfaw et al., 2012; Khonje et al., 2015). We utilize this method to estimate the effect of exposure to nutrition awareness on OFSP utilization in our study, and a non-parametric method (propensity score matching, PSM), to check on the robustness of obtained results.

### Estimation strategy

#### Endogenous switching regression

The endogenous switching regression estimation framework involves two stages, where the first stage is modelled as a Probit to understand the relationship between exposure to nutrition awareness and a number of household and village characteristics, while the second stage models the outcome variable, OFSP utilization conditional on a specified criterion function. In the first stage, a farmer participates in nutrition awareness interventions based on perceived benefits, ${D}^{*}$;

1 $$D_i^\ast=\beta X_i+\alpha Z_i+\mu_i,\;\mathrm{where}\;D_i=\left\{\begin{array}{c}1\;if\;d_i^\ast>0\\0\;otherwise\end{array}\right.$$

where ${D}_{i}^{*}$ is latent indicator of household *i*’s participation in nutrition awareness interventions; ${D}_{i}$ is the actual nutrition awareness indicator dummy (equal to 1 if the *i*^*t*h^ household was exposed to nutrition awareness and 0 if otherwise); ${X}_{i}$ is a vector of household and village characteristics, hypothesized to affect household *i*’s exposure to nutrition awareness; ${Z}_{i}$ is a vector of variables associated with exposure to nutrition awareness but not on OFSP utilization, used as selection instruments for identification of the model; $\beta$ and $\alpha$ are parameters to be estimated and $\mu$ is the error term. In the $Z$ vector, we include membership to groups and distance to main market as the selection instruments. Group membership signifies social capital and the likelihood of being exposed to nutrition awareness, while project intervention points are more likely to be found in the proximity of main village centers e.g., health clinics and schools. A simple falsification test shows that the two variables are jointly significant in explaining exposure to nutrition awareness [${\chi }^{2}$ = 18.86 (ρ = 000)] but not OFSP utilization [$F$=1.52 (ρ = 0.2210)] and therefore can be considered valid instruments.

In the second stage of the ESR framework, the second stage outcome equations are specified as:

2a $$\begin{array}{ccc}\mathrm{Regime }1:& {y}_{1i}={\beta }_{1}{X}_{1i}+{\upepsilon }_{1i}& \begin{array}{cc}\mathrm{if}& {D}_{i}=1\end{array}\end{array}$$

2b $$\begin{array}{ccc}\mathrm{Regime }2:& {y}_{2i}={\beta }_{2}{X}_{2i}+{{\upepsilon }}_{2i}& \begin{array}{cc}\mathrm{if}& {D}_{i}=0\end{array}\end{array}$$

where Regimes 1 and 2 refer to subpopulation exposed to nutrition awareness and not exposed, respectively; ${y}_{i}$ is the outcome variable indicator of OFSP utilization (number of days in the month OFSP is consumed in household i); $X$ is the vector of household *i* variables including sociodemographic, asset ownership and incomes.

The error terms in Eqs. (1), (2a) and (2b) are assumed to have trivariate normal distribution with zero mean and non-singular covariance matrix i.e.,

3 $$cov \left({\upepsilon }_{1i}, {{\upepsilon }}_{2i},{\mu }_{i}\right)=\left(\begin{array}{ccc}{\sigma }_{{\upepsilon }1}^{2}& & {\sigma }_{{\upepsilon }1\mu }\\ & {\sigma }_{{\upepsilon }2}^{2}& {\sigma }_{{\upepsilon }2\mu }\\ & & {\sigma }_{\mu }^{2}\end{array}\right)$$

where ${\sigma }_{{\upepsilon }1}^{2}$ and ${\sigma }_{{\upepsilon }2}^{2}$ are the variances of the error terms in the outcome Eqs. (2a) and (2b), respectively, and ${\sigma }_{\mu }^{2}$ is the variance of the selection Eq. (1) while ${\sigma }_{{\upepsilon }1\mu }$ and ${\sigma }_{{\upepsilon }2\mu }$ are the covariances between the selection and outcome equation error terms. Since the error term of the selection equation ($\mu$) is correlated with those of the outcome equations (${\upepsilon }_{1i}$ and ${\upepsilon }_{2i}$), the expected values of ${\upepsilon }_{1i}$ and ${\upepsilon }_{2i}$ conditional on the sample selection are non-zero;

4 $$\ E\left({\upepsilon }_{1i}|D=1\right)={\sigma }_{{\upepsilon }1\mu }\frac{{\phi} (\beta {X}_{i})}{{\sf \phi} (\beta {X}_{i})}={\sigma }_{{\upepsilon }1\mu }{\lambda }_{1i}\ \mathrm{and}$$

5 $$\ E\left({\upepsilon }_{2i}|D=0\right)=-{\sigma }_{{\upepsilon }2\mu }\frac{{\phi} (\beta {X}_{i})}{1-{\sf \phi} (\beta {X}_{i})}={\sigma }_{{\upepsilon }2\mu }{\lambda }_{2i}$$

where is $\phi$ the standard normal probability density function, $\phi$ is the standard normal cumulative density function; ${\lambda }_{1i}$ and ${\lambda }_{2i}$ are the inverse mills ratios calculated from the first stage equation and are plugged into the second stage outcome equations to correct for exposure and self-selection biases. The described ESR framework can be used to estimate the average treatment effect on a) the treated (ATT) and b) the untreated (ATU) by comparing the expected outcomes of households exposed to nutrition information and the non-exposed, in their counterfactual scenarios i.e., if the exposed were not exposed, and if the non-exposed were exposed. Following Di Falco et al. (2011), these conditional expectations for OFSP utilization can be defined as;

6a $$E\left({y}_{1i}|{D}_{i}=1\right)={\beta }_{1}{X}_{1i}+{\sigma }_{1\mu }{\lambda }_{1i}$$

6b $$E\left({y}_{2i}|{D}_{i}=0\right)={\beta }_{2}{X}_{2i}+{\sigma }_{2\mu }{\lambda }_{2i}$$

6c $$E\left({y}_{2i}|{D}_{i}=1\right)={\beta }_{2}{X}_{1i}+{\sigma }_{2\mu }{\lambda }_{1i}$$

6d $$E\left({y}_{1i}|{D}_{i}=0\right)={\beta }_{1}{X}_{2i}+{\sigma }_{1\mu }{\lambda }_{2i}$$

where Eqs. (6a) and (6b) represent the actual expectations from the observed sample of exposed and non-exposed households to nutrition awareness while (6c) and (6d) represent the counterfactual scenarios, respectively. Finally, the average effect of treatment (exposure to nutrition awareness) on the treated (ATT) is calculated as the difference between (6a) and (6c) while the average effect of the treatment on the untreated (ATU) is calculated as the difference between (6b) and (6d).

#### Propensity score methods

The ESR method discussed above may be sensitive to the validity of the chosen selection instruments. To check on the robustness of the results, we use the non-parametric propensity score matching method (PSM). The main tenet of PSM is to mimic a controlled experiment by using a predicted probability of exposure to treatment to create closely matched observations of treated and untreated units, to evaluate impact of the treatment. Using a counterfactual outcome framework (Wooldridge, 2002 Chapter 18), a household in our population of interest has two potential outcomes; the potential outcome for OSFP utilization when the household is exposed to the treatment i.e., nutrition awareness (${y}_{1}$), and the potential outcome when the household is not exposed (${y}_{0}$). Letting $d$ indicate exposure to nutrition awareness, where $d=1$ indicates that a household is exposed and $d=0$ indicates otherwise, the observed outcome can be written as

7 $$y=\left(1-d\right){y}_{0}+d{y}_{1}={y}_{0}+d({y}_{1}-{y}_{0})$$

of interest is the OFSP utilization outcome, with and without exposure to nutrition awareness, ${y}_{1}-{y}_{0}$.

Since we can only observe a household at one state i.e., either exposed to nutrition awareness or not, it is not possible to measure ${y}_{1}-{y}_{0}$ for a given household. Under the conditional independence assumption (Rosenbaum & Rubin, 1983), we can estimate the effect of treatment based on the observed random vectors $\left(\left({y}_{i},{d}_{i},{X}_{i}\right) i\dots n\right)$ using a randomly drawn sample from the population of interest, where $X$ is a list of covariates determining exposure to nutrition awareness, for household $i$. Under this assumption, the treatment status $d$ is independent of the potential outcomes ${y}_{1}$ and ${y}_{0}$ conditional on $x$, which allows for the matching of households in the treated and untreated groups based on similarities in the pretreatment observable characteristics (x);

8 $$p\left(x\right)=P\left(d=1|{\varvec{x}}\right)$$

where the function $p\left(x\right)$ is the response probability for treatment (probability of being exposed to nutrition awareness), also known as the *propensity score.* The average treatment effect (*ATE*) can then be estimated by taking expectation of the difference in outcomes with and without exposure to nutrition awareness (Dehejia & Wahba, 2002):

9 $$ATE=E\left\{{y}_{i1}-{y}_{i0}|d=1\right\}+E\left\{\left({y}_{i0}|d=1\right)-E\left({y}_{i0}|d=0\right)\right\}$$

where the first term on the RHS is the *average treatment effect on the treated* (ATET), or the OFSP utilization outcomes for the group that received nutrition awareness interventions, while the second term is the *average treatment effect on the non-treated* (ATENT), or the potential OFSP utilization outcomes for the group that did not receive nutrition awareness interventions, had they received these.

Several matching algorithms are used in the PSM literature including nearest neighbor matching (NNM) and kernel based matching (KBM) (Gitonga et al., 2020; Khonje et al., 2015). While these algorithms attempt to match the treated and non-treated units by the observable characteristics (x), the two groups may exhibit different distributions over these characteristics given the systematic differences due to the identified biases, leading to shrinking of the sample as control units that are not closely matched to treatment units are discarded during the analysis. Contrary to the propensity score based matching approaches, all observations are kept in the analysis in an approach using reweighting based on the propensity score ($p$), with each observation receiving the inverse of the probability of the treatment they actually got i.e., $1/{p}_{i}$ for the treated and $1/{1-p}_{i}$ for the untreated (see Rosenbaum and Rubin (1983)). A drawback to this approach is that it may lead to biased estimates if the selection equation is misspecified, as the inverse probability weights are solely based on the estimated propensity score. The “doubly robust” approaches, such as the inverse probability weighting regression adjustment (IPWRA), addresses these limitations by combining the inverse probability weighting above with a regression adjustment meant to correct for misspecification in the outcome equations (see Emsley et al. (2008)). We compare the results from the re-weighted propensity score-based *treatrew* estimation with those from IPWRA, both as a check for the robustness of results obtained from the ESR method discussed before.

## Data

### Data sources and sampling

Primary data was collected in the months of May–July 2021, slightly over a year after the start of nutrition interventions in the study areas of Garissa, Tana River, Isiolo and Baringo counties. Quantitative data was collected from households using a semi-structured questionnaire, while qualitative data was collected from county nutrition and agricultural extension officers, as well as community members and leaders, using focus group discussions (FGDs) and key informant interviews (KII’s). In this study, we use the household survey data to analyze the effect of nutrition awareness on OFSP utilization, and the qualitative data to further understand the insights behind observed results. These insights are also used to further ground the attribution of observed effect of nutrition awareness on OFSP utilization to interventions led by CIP-WFP.

To identify households for the household survey, a multistage sampling procedure was used; in the first step, the four counties of study were purposively selected based on their sweetpotato production potential, determined with the help of partners from the national and county agricultural offices. Next, WFP field officers working with county agricultural officers assisted in compiling a list of all sweetpotato producing areas within these counties. The list also indicated administrative areas (locations, sub-locations and villages) where CIP-WFP interventions on scaling out OFSP were being promoted. This formed a basis for stratifying the clusters for inclusion in the study, with villages stratified as either areas of intervention or otherwise. Finally, random-proportionate to size sampling was utilized to select 550 households spread across 51 villages in 26 locations of the four counties.

### Description of key variables

In this section, we describe the various variables used in the study, as well as provide the descriptive statistics for these.

#### Nutrition awareness

**Fig. 1:**
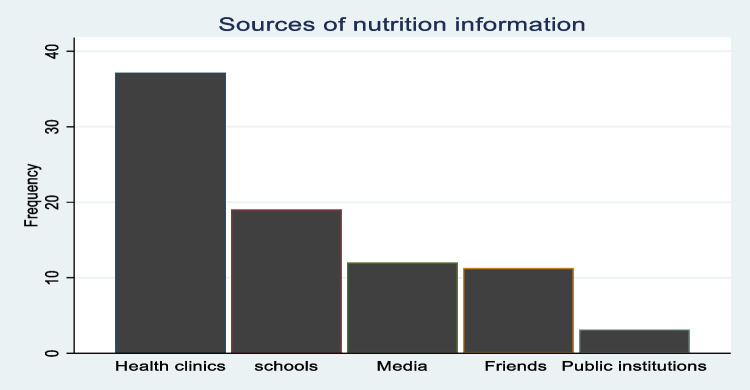
Sources of nutrition information

This is the main predictor (*treatment)* variable in our study. To elicit this, respondents were asked if they had received any information about Vitamin A and its nutritional importance, as well as knowledge of OFSP and its benefits in boosting Vitamin A intake. The nutrition awareness variable thus obtained is a composite variable and could be achieved through any of the promoted CIP-WFP interventions in the area. Given that these interventions in the study region are channeled through various change agents, including health clinics for pregnant mothers, schools, and public extension (see Fig. 1), it may not be feasible to directly elicit participation in a single CIP-WFP intervention, as a treatment variable.

For example, information flow through participating beneficiaries may diffuse from the individual to other household members and friends, as well as spillover to neighboring households. The reasonable assumption made is that observed nutrition awareness is due to any of the CIP-WFP intervention arms, either through direct beneficiaries or through community spillovers.

#### OFSP utilization

As an indicator of OFSP utilization at the household level, we use consumption of OFSP by members of the household vulnerable to VAD, whether the household accessed OFSP roots and/or puree from own production, market purchases or through food aid. To do this, we asked respondents about number of times OFSP was consumed in their households in the previous one month. Specifically, this was asked for the household members most vulnerable to VAD i.e., children less than 5 years old and pregnant and/or lactating mothers. Given the low numbers of pregnant and lactating mothers in the sample and in line with extant literature (Wilde et al., 1999), this category was revised to refer to female household members of child-bearing age.

The main outcome variable is therefore a continuous variable of the number of days in the previous one month that any of these two member groups in a household (i.e., women of child-bearing age and children under 5 years of age) had consumed OFSP. This redefinition allowed for an analysis using 539 observations out of the 550 interviewed households (see Table 1).

**Table 1:** Descriptive statistics

Variable	Exposed to nutrition awareness (n = 368)	Non-exposed to nutrition awareness (n = 166)	Test for difference in means		Overall (n = 534)	
	Mean	Mean	Mean	Std error	Mean	SD
Exposure to nutrition awareness (%)					68.9	-
*Outcome variables*						
Consumed OFSP (1 = Yes)	21.5	10.2	11.2***	0.032	17.7	-
Times consumed OFSP last one month	2.06	0.54	1.52***	0.386	1.59	3.28
*Household head characteristics*						
Female-headed (%)	42	40	2.0	-	41.6	-
Education	7.0	5.3	-1.7***	0.44	6.5	4.8
Age	45	50	4.6***	1.29	46	13.9
*Household characteristics*						
Group membership	26.6	15.2	-2.91***	0.004	23.0	-
Access to extension	15.2	7.2	-2.33**	0.020	12.9	-
Distance to market	55.2	66.2	10.84**	4.81	58.9	51.7
Pregnant woman in household	20.9	14.2	-1.77*	0.077	18.7	-
*Fixed effects*						
Baringo county					34.9	-
Tana river county					17.9	-
Garissa county					18.4	-
Isiolo county					28.7	-

*** p < 0.01, ** p < 0.05, * p < 0.1

#### Other explanatory variables

Variables hypothesized to be important in explaining exposure to nutritional information include sociodemographic variables such as sex, age and education of household head, group membership, proximity to markets and access to extension. Consistent with previous literature on exposure to- and adoption of- technologies, female-headed households are hypothesized to be less exposed to nutritional information while higher educated heads are more likely to exposed to nutritional information. A quadratic relationship between age and exposure to nutritional information is expected, with older household heads more exposed to nutritional information until a maximum point where increase in age induces less exposure to nutritional information.

Following literature on technology diffusion theory, membership to groups is expected to be positively correlated to exposure to nutritional information, similar to access to extension advice. On the other hand, a negative relationship between exposure to nutritional information and proximity to markets is expected, with households far away from markets expected to be less exposed to nutritional information. This is since the main intervention points for dissemination of nutrition awareness are health clinics and schools, which are also likely to be found in market centres. We also include an indicator variable of whether a household has a pregnant woman at the time of the study, as one of the entry points of the WFP-CIP interventions was dissemination of nutritional information through trained pre- and post-natal clinic workers, as trainer of trainers (ToTs). This variable is therefore hypothesized to be positively correlated with exposure to nutrition awareness.

## Results

Results of the study are presented in this section. First, we present results from a Probit estimation of the determinants of exposure to nutrition awareness, followed by those on the effect of exposure to nutrition awareness on OFSP utilization. For the latter, we first present results from the endogenous switching regression (ESR), followed by those from two estimators based on the propensity score matching (PSM) method, the *treatrew* procedure following (Cerulli, 2014) and the inverse probability weighting regression adjustment (IPWRA).

### Determinants of exposure to nutrition awareness

Results on the determinants of nutrition awareness are presented in Table 2. Consistent with existing literature, female-headed households were shown to be less likely to be exposed to nutrition information. This is despite the CIP-WFP intervention strategy of targeting women, especially the pregnant and lactating mothers using the health clinics approach. One explanation to this puzzle is that women from female-headed households, which have been shown by literature to be resource poor (Felker-Kantor & Wood, 2012), are less likely to attend pre- and ante-natal clinics in these regions. As a result, while the targeting strategy manages to get many women in the intervention, these are likely to come from households that are less resource and time constrained, and which are mainly male headed. There is need to therefore revise the targeting strategy to include women who are less likely to attend such clinics, for inclusive impacts of the interventions. Similarly, heads of households with higher formal education had a higher likelihood of being exposed to nutrition awareness. In a region where much of the population has low levels of formal education, interventions aimed at scaling out utilization of OFSP need to be sensitive to education-related barriers.

**Table 2:** ESR selection equation estimates on the determinants of exposure to nutrition awareness

VARIABLES	Vitamin A & OFSP awareness
Female household head (1 = Yes)	-0.370**
	(0.183)
Household head education	0.0981***
	(0.0201)
Household head age	0.0163
	(0.0509)
Household head age-squared_	-0.000200
	(0.000514)
Group membership	0.362**
	(0.181)
Extension access	0.897**
	(0.445)
Distance to market	-0.00360**
(Walking minutes)	(0.00176)
Pregnant woman in household	0.912***
	(0.319)
Garissa county	0.409**
	(0.181)
Isiolo county	0.658**
	(0.258)
Tana River county	0.948***
	(0.273)
Constant	-0.263
	(0.726)
Observations	528

Standard errors in parentheses.

*** p < 0.01, ** p < 0.05, * p < 0.1

Institutional related factors such as market and extension access were also found to be significant in explaining exposure to nutrition awareness. Specifically, households with access to extension service were more likely to be exposed to nutrition awareness, while those further away from main markets were less likely to be exposed. As mentioned before, the main intervention points for nutrition awareness are health clinics and schools, which are also likely to be found in market centres in the intervention areas. This would explain the finding of proximity to such centres and nutrition awareness. Incidentally, households further away from markets are more likely to be vulnerable to malnutrition, as nutritional diversity for these households depends more on own production. Deepening access to nutrition awareness through enhancing other interventions such as the support to village level traders may thus further unlock OFSP utilization through spread of nutrition awareness and access to OFSP roots. Similarly, the CIP-WFP suite of intervention packages includes training of public extension officers on nutrition awareness, for the onward dissemination of the same to farming households. Targeting local-based extension agents, at lower administrative units and those close to farmers will therefore achieve higher adoption of OFSP for producing households.

### Impact of nutrition awareness on OFSP utilization

#### Results based on the Endogenous switching regression method

We first present results from the ESR-based average treatment effects of exposure to nutrition awareness on OFSP utilization under actual and counterfactual conditions (Table 3). The ESR estimates of determinants to OFSP utilization are presented in Table 5 in Appendix but are not discussed in this paper for brevity. It is however interesting to note that the estimated coefficient, ${\rho }_{i}$, of the correlation between the error term of the selection equation, ${\mu }_{i}$, and that of the outcome equation for the sub-sample exposed to nutrition awareness, ${\upepsilon }_{1i}$, is statistically significant from zero. We may thus reject the null hypothesis of absence of sample selectivity bias and justify the use of the ESR method to correct for this.

**Table 3:** Results from endogenous switching regression

	Exposed to nutrition awareness	Non-exposed to nutrition awareness	Treatment effects
Households with nutrition awareness (ATET)	2.2 (0.079)	0.8 (0.055)	1.4 (0.096) ***
Households without nutrition awareness (ATENT)	1.6 (0.112)	0.6 (0. 0.078)	1.0 (0.137) ***

Standard errors in parentheses.

*** p < 0.01, ** p < 0.05, * p < 0.1

Controlling for other covariates determining utilization of OFSP and the selection bias from both observed and unobserved heterogeneity, exposure to nutrition awareness led to significant improvements to OFSP utilization (proxied by number of days vulnerable members of a household consumed OFSP within the previous 30 days). Specifically, households exposed to nutrition awareness had on average 2.2 days of OFSP consumption within the previous 30 days, compared to the counterfactual condition of 0.8 days, had they not been exposed. This gives an overall average effect of 1.4 days of OFSP consumption, with exposure to nutrition awareness. On the other hand, households that were not exposed to nutrition awareness would benefit by having about 1.6 days of OFSP consumption had they been exposed, compared to the actual 0.6 days of OFSP consumption with non-exposure, giving an overall effect of a day of OFSP consumption on the non-exposed population.

#### Results from propensity score weighting

**Fig. 2:**
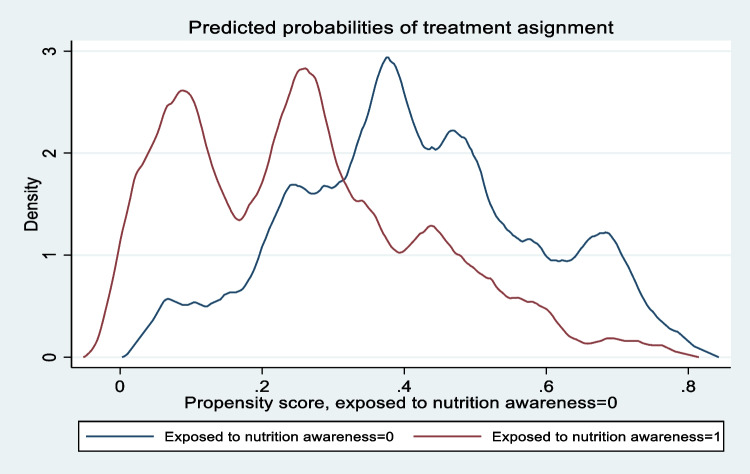
Test for the overlap assumption

This section presents results from the re-weighted propensity score and the inverse probability weighting regression adjustment (IPWRA) methods. A balancing test conducted after the estimation of the weighted propensity scores show that we fail to reject the null hypothesis that the covariates are balanced after weighting [${\chi }^{2}$ = 2.09 (ρ = 0.990)], and therefore the balancing condition was met. Similarly, a test for the overlap property shows the overlap assumption is not violated; neither of the estimated densities are close to 0 or 1, with much of the respective masses found in regions of overlap (Fig. 2).

Next, we present results on the effect of exposure to nutrition awareness on OFSP utilization. The first column in Table 4 represent results from the re-weighted propensity score using the *treatrew* command with bootstrapped standard errors, while those in the second column are from the inverse probability weighting regression adjustment (IPWRA) estimation.

**Table 4:** Results from re-weighted propensity score and inverse probability weighting regression adjustment

VARIABLES	Number of days consumed OFSP last one month	
	Re-weighted propensity score	Inverse Probability Weighting Regression Adjustment (IPWRA)
	BS SE	
ATE	1.25***	2.41**
	(0.395)	(1.235)
ATET	1.137***	3.11*
	(0.471)	(1.867)
ATENT	0.97***	-
	(0.390)	

Standard errors in parentheses.

*** p < 0.01, ** p < 0.05, * p < 0.1

Results from the re-weighted propensity score (*treatrew*) estimation are closest to those obtained from the ESR estimation and show that on average, households exposed to nutrition awareness have on average about 1.3 days of OFSP consumption in a month, compared to similar households not exposed to nutrition awareness. On their part, households not exposed to nutrition awareness would have had about a day of OFSP consumption in the last one month had they been exposed. The average effects are even higher with the IPWRA estimation, with the effect of exposure to nutrition awareness being about 3.1 days of OFSP consumption on average. The positive and highly significant results of the effect of exposure to nutrition awareness on OFSP utilization are robust across the various estimation methods and point to the crucial role played by awareness creation interventions in enhancing OFSP utilization. These results are also consistent with others on demand side interventions aimed at enhancing OFSP adoption (Adekambi et al., 2020) and consumption (Just et al., 2022).

## Discussion and conclusions

Vitamin A deficiency (VAD) continues to afflict vulnerable populations in lower- and middle-income countries (LMICs), with those in fragile environments being affected the most. This study aims at providing early evidence on the effects of interventions led by the World Food Program (WFP) and the International Potato Center (CIP) through a partnership to enhance food and nutritional security in arid and semi-arid regions of Kenya. The study uses cross-sectional data and robust impact evaluation methodologies to understand the effect of raising nutrition awareness on utilization of orange fleshed sweetpotato (OFSP), a micronutrient (Vitamin A) rich biofortified crop. Further, the study assesses some of the intervening factors that determine exposure to nutrition awareness, with a view of identifying lessons to guide ongoing interventions to achieve biofortification at scale and inclusive impacts in the region.

Key factors determining exposure to nutrition awareness identified in the study include household characteristics like gender and education, and institutional factors like market and extension access. Consistent with the literature, female-headed households are less likely to be exposed to nutrition awareness, despite the CIP-WFP intervention strategy of targeting women, especially the pregnant and lactating mothers using the health clinics approach. There is need to therefore rethink this strategy, as participating women in these interventions are more likely to be drawn from households that are less resource and time constrained, which are mainly male-headed. A more nuanced gendered strategy will ensure women from the most vulnerable households and who are unable to be drawn in the sample of women attending health clinics are also reached by the interventions. Similarly, household heads with lower levels of formal education are shown to have a lower likelihood of accessing nutritional information. Nutrition awareness interventions, such as those disseminated through the media, need to take this into account to reach these lowly-educated households, who are also some of the most vulnerable to malnutrition. Programming of nutrition information could for example be more focused on using local languages, as well as be accompanied by demonstrations, to improve on salience.

Distance to main markets is negatively correlated with access to nutrition information. This could be driven by the fact that most intervention points, such as health clinics, are found near main markets. Deepening other intervention areas, such as support to traders at village level markets, would bring nutrition information and access to OFSP closer to marginalized areas and enhance OFSP utilization. Similarly, access to extension advice improves the likelihood of access to nutrition information, which is also likely driven by the CIP-WFP intervention arm of training public extension officers on nutrition awareness, for onward dissemination to farming households. An integrated approach of targeting extension agents both at county and lower administrative levels, e.g., location levels, would ensure nutrition information is brought closer to vulnerable households who are far from county extension offices. This is especially important given the current agricultural extension policy in the country that is more demand- rather than supply- driven.

The results also identify clear positive effects of access to nutrition awareness on OFSP utilization. These results, though modest (on average about a day and half of OFSP utilization in a 30-day period), are highly significant and clearly show the potential of the CIP-WFP interventions in enhancing biofortification in the ASAL regions of Kenya. Given that these interventions are still at nascent stage, the results are quite encouraging as to the success potential of such interventions in enhancing OFSP utilization in the region. Further, considering that this analysis focuses solely on interventions aimed at raising nutrition awareness, combining such with other interventions meant to mitigate demand-side constraints (for example behavioral constraints) will result in higher OFSP utilization. These could further be combined with supply-side enablers, for example, higher accessibility to OFSP vines at affordable prices, WFP’s cash transfer program for vulnerable households and linkages to OFSP markets, and support for greater market spread of OFSP roots for increased accessibility to the produce to both producing and non-producing households.

## Appendix

**Table 5:** Endogenous switching regression estimates for OFSP consumption in the ASALs of Kenya

	Number of days household consumed OFSP		
VARIABLES	Non-exposed to nutrition awareness	Exposed to nutrition awareness	Selection (1/0)
Household head sex	0.209	0.0639	-0.370**
	(0.268)	(0.495)	(0.183)
Household head education	0.0110	-0.172***	0.0981***
	(0.0421)	(0.0620)	(0.0201)
Household head age	-0.0630	-0.0951	0.0163
	(0.0852)	(0.103)	(0.0509)
Household head age-squared_	0.000889	0.000920	-0.000200
	(0.000922)	(0.00118)	(0.000514)
Group membership	-	-	0.501*
			(0.264)
Distance to market	-	-	-0.00360**
(Walking minutes)			(0.00176)
Extension access	1.641	0.268	0.897**
	(2.064)	(0.757)	(0.445)
Pregnant woman in household	-0.592	-1.823***	0.912***
	(0.557)	(0.703)	(0.319)
Access to off-farm income	0.412	-0.239	-
	(0.409)	(0.488)	
Total land owned (acres)	-0.00441	0.0584***	-
	(0.0137)	(0.0213)	
Asset index	-18.40	-0.177**	-
	(12.77)	(0.0717)	
County controls	0.840***	0.635**	0.381***
	(0.281)	(0.281)	(0.135)
${\sigma }_{i}$	0.381**	1.248***	
	(0.163)	(0.114)	
${\uprho }_{i}$	-0.0447	-0.512**	
	(0.289)	(0.253)	

Robust standard errors clustered at the village level in parentheses. ${\sigma }_{i}$ denotes the square-root of the variance of the error terms ${\upepsilon }_{ji}$ in the outcome Eqs. (2a) and (2b), respectively; ${\uprho }_{i}$ denotes the correlation coefficient between the error term ${\mu }_{i}$ of the selection Eq. (1) and the error term ${\upepsilon }_{ji}$ of the outcome Eqs. (2a) and (2b), respectively
